# Beta-HPV 5 and 8 E6 Promote p300 Degradation by Blocking AKT/p300 Association

**DOI:** 10.1371/journal.ppat.1002211

**Published:** 2011-08-25

**Authors:** Heather L. Howie, Jennifer I. Koop, Joleen Weese, Kristin Robinson, Greg Wipf, Leslie Kim, Denise A. Galloway

**Affiliations:** 1 Division of Human Biology, Fred Hutchinson Cancer Research Center, Seattle, Washington, United States of America; 2 Molecular and Cellular Biology Program, University of Washington, Seattle, Washington, United States of America; University of Michigan, United States of America

## Abstract

The E6 oncoprotein from high-risk genus alpha human papillomaviruses (α-HPVs), such as HPV 16, has been well characterized with respect to the host-cell proteins it interacts with and corresponding signaling pathways that are disrupted due to these interactions. Less is known regarding the interacting partners of E6 from the genus beta papillomaviruses (β-HPVs); however, it is generally thought that β-HPV E6 proteins do not interact with many of the proteins known to bind to α-HPV E6. Here we identify p300 as a protein that interacts directly with E6 from both α- and β-HPV types. Importantly, this association appears much stronger with β-HPV types 5 and 8-E6 than with α-HPV type 16-E6 or β-HPV type 38-E6. We demonstrate that the enhanced association between 5/8-E6 and p300 leads to p300 degradation in a proteasomal-dependent but E6AP-independent manner. Rather, 5/8-E6 inhibit the association of AKT with p300, an event necessary to ensure p300 stability within the cell. Finally, we demonstrate that the decreased p300 protein levels concomitantly affect downstream signaling events, such as the expression of differentiation markers K1, K10 and Involucrin. Together, these results demonstrate a unique way in which β-HPV E6 proteins are able to affect host-cell signaling in a manner distinct from that of the α-HPVs.

## Author Summary

Human papillomaviruses (HPVs) are a family of more than 100 different viruses that cause a wide range of pathologies, from benign warts to cervical cancer. One subgroup of HPVs, the beta-HPVs, have recently become a topic of interest due to their potential involvement in squamous cell skin cancer. However, unlike the HPVs involved in cervical cancer, little is known with regards to how the beta-HPVs may facilitate cellular changes that would allow cancerous lesions to develop. Here we have identified a host-cell protein, p300, which interacts strongly with the E6 oncoprotein from two beta-HPVs, HPV 5 and HPV 8. We show that this interaction subsequently blocks another cellular protein, AKT, from binding to and stabilizing p300. By blocking this association, p300 is targeted for degradation, and thus is present in lower amounts than in normal cells. Importantly, because p300 is involved in numerous cell processes such as DNA repair, cell growth, and differentiation, the potential for E6 disrupting a number of cellular signaling pathways is vast. Taken together, our findings shed new light on how the beta-HPVs may facilitate carcinogenesis.

## Introduction

Human papillomaviruses (HPVs) are a large family of DNA tumor viruses that infect both cutaneous and mucosal epithelium, and lead to a range of pathologies, from benign papillomas to cancerous lesions. Over 130 different HPV types have been identified and divided into a number of genera based on DNA sequence homologies [1]. The best-studied HPVs are those of the alpha genus (α-HPVs), and include both low-risk (HPV types 6 and 11) and high-risk (HPV types 16 and 18) viruses. Low-risk HPVs have been most often associated with genital warts and non-cancerous papillomas, whereas the high-risk HPVs have been shown to be the etiologic agent in cervical cancer, as well as other anogenital carcinomas and a subset of head and neck cancers [2], [3]. Recently, another group of HPVs, the beta-HPVs (β-HPVs) have become the subject of interest due to their possible involvement in squamous cell skin carcinoma (SCSC) [4], [5], [6], [7].

All HPVs encode the E6 and E7 oncoproteins, which are responsible for numerous physiological changes within the infected host cell [reviewed in 8,9]. However, E6 and E7 proteins differ functionally among different HPV genera, species and types. In the α-HPV genus, some E6 functions are conserved between both high- and low-risk HPV types, including association with the E3 ubiquitin ligase E6AP [10], [11], [12], [13] and degradation of the pro-apoptotic protein Bak [14], [15]. Conversely, many E6 functions are manifested primarily by high-risk α-HPVs, including the activation of telomerase [16], [17], [18] and the degradation of a number of proteins including p53 [19] and PDZ domain containing proteins such as hDlg [20], [21], hScrib [22], MAGI [23], [24], MUPP1 [25], and PTPN3 [26], [27]. Not surprisingly, some of these functional differences can be attributed to variations in the E6 amino-acid sequence [24], [28]. For example, only high-risk α-HPVs harbor a PDZ domain, which explains why they are able to associate with PDZ proteins and low-risk E6 proteins are not.

While the functions of E6 from both the high- and low-risk α-HPVs have been well studied, little is known about how E6 from β-HPVs contribute to viral pathogenicity. Recent studies have demonstrated that like α-HPV E6, some β-HPV E6 proteins are capable of activating telomerase, and interacting with Bak and E6AP [29], [30], however other well-documented E6 functions are not conserved between these two genera. For example, as β-HPV E6 proteins (like the low-risk α-HPV E6 proteins) lack a PDZ domain, they are unable to interact with and disrupt key polarity signaling pathways, as do high-risk α-HPV E6 proteins. Moreover, while some β-HPV E6 proteins, like 38E6, have been shown to perturb p53 signaling through transcriptional activation of deltaNp73 [31], [32], most β-HPV E6 proteins are unable to bind p53, making it unclear as to whether they are capable of inactivating p53 signaling; a crucial step in carcinogenesis for the high-risk α-HPVs. It is unclear what other protein interactions occur between the β-HPV E6 proteins and host-cell proteins, the subsequent signaling pathways that may be disrupted due to these interactions, and the role these interactions may play in the development of SCSC.

One protein that has garnered interest due to its ability to interact with E6 from both α- and β-HPVs, Bovine Papillomavirus (BPV), and Cottontail Rabbit Papillomavirus (CRPV) is the histone acetyltransferase p300 [33],[34],[35],[36],[37],[38]. p300 is a central hub in numerous signaling pathways, and consequently has been shown to associate with over 100 different proteins [Reviewed in 39,40], thus the potential for E6 to disrupt important signaling pathways via association with p300 is vast. E6 from high-risk α-HPVs has been shown to bind to three distinct regions of p300; the C/H1 domain, the C/H3 domain and the C-terminus, and disrupt important p300-dependent signaling events such as p53 and NFΚB transactivation [34], [36], [38]. Conversely, association of E6 from low-risk α-HPVs appears to be confined to the C/H1 region, and conflicting evidence has been reported as to whether or not this association alters p300-mediated signaling [34], [36], [38]. Interestingly, as seen with E6 from the high-risk α-HPVs, BPV 1E6 and β-HPV 8E6 have both been shown to bind to the C/H3 region of p300, while 8E6 is also capable of binding to the C/H1 and C-terminal domains [33], [35]. This association has been shown to attenuate p53 transactivation in the case of BPV 1E6 [35], however it is unknown if 8E6 causes the same effect. Most recently, CRPV E6 and β-HPV 38E6 were both shown to interact with full-length p300, and these interactions also attenuated p53 signaling [37]. As can be seen, E6 proteins from different HPVs associate with different domains of p300 with different effects. Likewise, at least two distinct regions of E6 have been implicated in this interaction; the region encompassing the second zinc-finger domain corresponding to aa 100-147 of 16E6 [33], [34], [38], and a more N-terminal region corresponding to aa 75–84 of 38E6 [37]. The region of 8E6 that bound p300 was mapped to residues 132–136 [33]. Thus, even when associating with the same protein, E6 from different HPVs handle this interaction in a unique manner.

In order to better understand what signaling pathways may be altered by β-HPV E6, we set out to identify host-cell proteins that might interact with β-HPV E6 proteins. GST-pulldowns were performed using recombinant GST-E6 proteins from a number of β-HPVs and lysates from human foreskin keratinocytes (HFKs) The isolated complexes were then analyzed by mass spectrometry. Here we describe p300, as a protein that interacts with all of the E6 proteins tested, but with different strengths and in turn different consequences. Importantly, we found that β-HPV 5 and 8E6 bound to p300 very strongly, which in turn led to the proteasomal mediated degradation of the p300 protein. We provide evidence that degradation is mediated by E6 occluding the AKT-phosphorylation site on p300, which, when phosphorylated, maintains the stability of p300 within the cell. Finally, we demonstrate that lower p300 levels in HPV 5 and 8E6 expressing cells, in turn, affects normal p300-dependent signaling pathways.

## Results

### The E6 proteins from HPV types 5, 8, 38 and 16 associate with p300

In order to identify the cellular interacting partners of various β-HPV E6 proteins, N-terminally GST-tagged E6 proteins from β-HPV types 5, 8, and 38 and α-HPV type 16 (as a control) were purified and incubated with whole cell lysates from primary human foreskin keratinocytes (HFKs). Interacting proteins were extracted using glutathione beads, and identified by mass spectrometry following SDS-PAGE separation. This resulted in the identification of hundreds of potential E6 interacting proteins, including those that interacted with all of the purified E6 proteins tested, and those that interacted with only select E6 proteins (see Table S1). Importantly, we were able to identify E6AP, hScrib, and hDlg peptides with purified 16E6, thus validating the efficacy of our pulldown and identification protocol.

**Figure 1:**
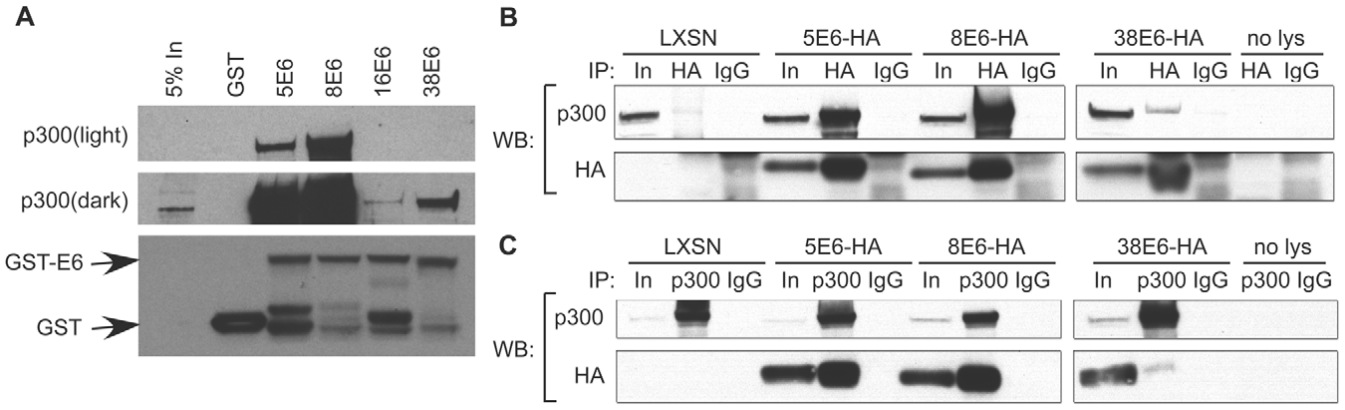
E6 from α and β HPVs bind p300 with different strengths. (A) Representative immunoblot showing the levels of p300 co-precipitating with various GST-tagged E6 proteins. Equal levels of input GST-E6 are demonstrated by the GST immunoblot in the lower portion of the figure. (B) HFKs expressing E6-HA were immunoprecipitated with a HA antibody (HA) or mouse Immunoglobulin G (IgG) as a negative control and immunoblotted with a p300 and HA antibody. (C) Identical cell lysates were immunoprecipitated with a p300 antibody (p300) or IgG control and immunoblotted with a p300 and HA antibody. Input is equal to 5% of total protein lysate.

Of particular interest, 9–12 unique peptides corresponding to the histone acetyltransferase p300 were identified in the lysates incubated with either HPV5 or 8E6. Four peptides from the related protein, CBP, were also identified from lysates incubated with 8E6. Surprisingly, no p300 or CBP peptides were identified from lysates incubated with HPV 16 or 38E6, even though an association with p300 has previously been shown with E6 from these HPV types [34], [36], [37], [38]. To verify the interaction with p300, lysates from parallel GST-pulldown assays were subjected to immunoblot analysis, in which membranes were probed using an antibody for p300. Interestingly, p300 co-immunoprecipitated with each of the β-HPV types tested, as well as with α-HPV type 16E6 (Figure 1A). However, the interaction between p300 and E6 from β-HPV types 5 and 8 was much stronger than that between β-HPV 38E6 or α-HPV 16E6, as evidenced by the relative signal intensities on the immunoblot. Importantly, differences in the magnitude of the interaction of E6 and p300 among different types has not been previously reported and suggests that some E6 proteins may interact with p300 in a unique manner to affect p300 functions.

To verify the interaction between p300 and E6 *in vivo*, C-terminally HA-tagged E6 proteins were expressed in HFKs, and E6 expression verified by immunoblot (Figure 1B “In” lanes). We were unable to express 16E6-HA at sufficiently high levels needed for comparative binding assessment, therefore only 5-, 8- and 38E6-HA are shown in Figure 1B and C. E6-HA expressing cell lysates were then incubated with an anti-HA antibody to immunoprecipitate complexes that bound to E6. Subsequent immunoblotting demonstrated that 5, 8 and 38 E6 all interacted with p300 in vivo, and verified that the interaction of 5 and 8E6 with p300 was many times stronger than that seen with 38E6 (Figure 1B). Co-immunoprecipitations were repeated in the reverse direction, by pulling down with an anti-p300 antibody, followed by immunoblot analysis against HA. Once again, p300-E6 interactions were seen in 5, 8 and 38E6-HA expressing cells, with 5, and 8 E6 interactions being the highest (Figure 1C). Taken together, these data demonstrate that p300 interacts with the E6 protein from multiple β-HPV types. Moreover, the observed differences in the magnitude of this interaction suggests there may be different consequences to p300-mediated signaling events in cells expressing each of these E6 proteins.

### β-HPV 5 and 8E6 degrade p300 in a proteasomal-dependent, but E6AP-independent manner

**Figure 2:**
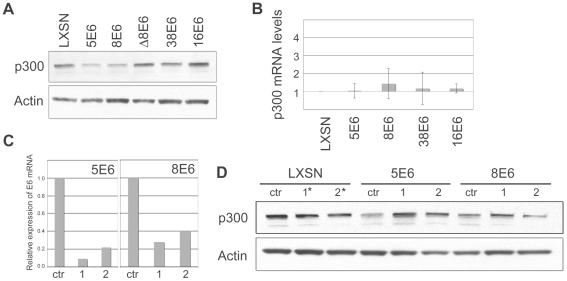
β HPV 5 and 8E6 binding to p300 leads to p300 degradation. (A) Representative immunoblot showing the levels of p300 in LXSN and E6-expressing HFKs. Actin is shown as a loading control. (B) p300 mRNA levels in LXSN and E6-expressing HFKs. Relative levels of p300 mRNA were calculated using the ΔΔCT method with GAPDH to normalize mRNA levels within each sample. Values shown are the mean fold-change in each sample compared to the LXSN vector control. Error bars represent the standard deviation for each sample (n = 3). (C) 5E6 and 8E6 mRNA levels following transfection of E6 siRNA. Relative levels of 5 and 8E6 mRNA were calculated using the ΔΔCT method with GAPDH to normalize mRNA levels within each sample. Values shown are the mean fold-change in each sample for one representative experiment. (D) Representative immunoblot showing the levels of p300 in LXSN, 5E6 and 8E6 expressing cells following transfection with siRNAs specific for each E6. Actin is shown as a loading control. siRNA #1* and #2* in LXSN cells represent a 50/50 mixture of 5E6 siRNA #1 and 8E6 siRNA #1, and 5E6 siRNA #2 and 8E6 siRNA #2 respectively.

Given the observation that 5 and 8E6 interacted with p300 to such a great extent, we wished to determine the consequences of this interaction in E6 expressing cells. Importantly, the interaction of 16E6 with p300 has previously been shown to alter a number of signaling pathways, including p53 activation [34], [36], [38]. Moreover, while 8E6 has previously been shown to interact with p300, the consequences of this interaction with respect to host-cell signaling has not been examined [33]. We first wished to determine if E6 expression had an effect on p300 protein levels within the cell, as the stability of many proteins known to interact with high-risk E6 is altered. Cell lysates from vector control LXSN, or E6-expressing HFKs were analyzed by immunoblotting for p300. Surprisingly, levels of p300 protein were decreased in cells expressing 5 and 8E6 as compared to cells expressing either 38E6, 16E6 or vector alone (Figure 2A). Importantly, expression of a p300-binding deficient 8E6 mutant harboring a 5aa deletion between residues 132–136 (designated here as Δ8E6, [33], see Table S2 for E6 alignment at these residues) did not lead to decreased levels of p300, indicating that association between the two proteins is necessary for this response. Real-time RT-PCR demonstrated that levels of p300 mRNA remain unchanged in each of these cells (Figure 2B), suggesting that the lower levels of p300 seen in 5 and 8E6 expressing cells may be due to degradation of the p300 protein. To ensure that 5E6 or 8E6 expression was required for p300 degradation, siRNAs for each respective E6 protein were transfected into LXSN, 5E6 or 8E6 expressing cells, and lysates harvested for RNA and protein. E6 mRNA knockdown was verified by real-time RT-PCR for the respective E6 (Figure 2C). Examination of p300 protein levels in these cells revealed that p300 protein levels increased upon E6 knockdown for both siRNAs targeting 5E6 and one siRNA targeting 8E6 (Figure 2D). The second 8E6-specific siRNA did not lead to increased p300 expression, but was also the siRNA with the least efficient knockdown of E6, indicating a possible dose-response effect of E6 expression toward p300 degradation (Figure 2C).

**Figure 3:**
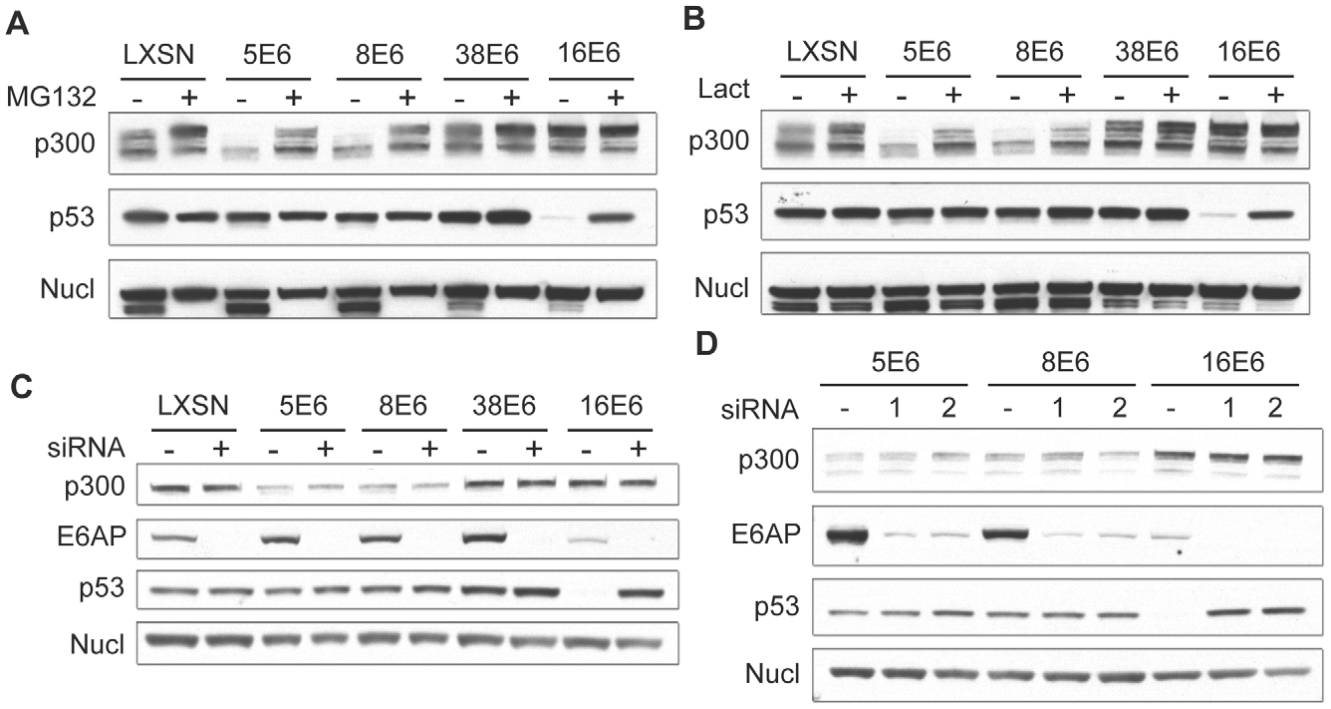
β HPV 5 and 8E6 degrade p300 in a proteasomal-dependent, E6AP-independent manner. (A) LXSN and E6-expressing HFKs were treated with DMSO (−) or MG132 (+) 2 hr prior to harvesting cell lysates. Lysates were then analyzed by immunoblot for p300, p53 and nucleolin (control). (B) LXSN and E6-expressing HFKs were treated with DMS0 (−) or Lactacystin (+) 2 hr prior to harvesting cell lysates. Lysates were then analyzed by immunoblot for p300, p53 and nucleolin. (C) E6AP was knocked down by transfection of LXSN or E6-expressing HFKs with a pool of 4 siRNAs targeting E6AP (+) and compared to cells mock treated by transfection with a pool of 4 non-targeting siRNAs (−). 72 hr post transfection, lysates were harvested and analyzed by immunoblot for p300, E6AP, p53 and nucleolin (control). (D) E6AP was knocked down using 2 individual siRNAs from the above pool (1 and 2) and compared to cells mock treated with 1 individual non-targeting siRNA (−). 72 hr post transfection, lysates were harvested and analyzed by immunoblot for p300, E6AP, p53 and nucleolin (control).

High-risk E6 proteins such as 16E6 are well known for their ability to promote protein degradation in a proteasomal-dependent manner. To test if the decreased levels of p300 protein observed in 5 and 8E6 expressing cells was due to proteasomal degradation, proteasomal inhibitors MG132 and Lactacystin were used. LXSN control and E6 expressing cells were incubated with either MG132 or Lactacystin for 2 hrs prior to cell lysis. Cell lysates were then harvested and analyzed by immunoblot to determine the levels of p300 protein (Figure 3A and 3B). As a control, p53 levels were also analyzed, as p53 is known to be degraded by 16E6 in a proteasomal-mediated fashion [19]. In the absence of inhibitor, p53 was degraded in 16E6 expressing cells; however, in the presence of either MG132 or Lactacystin, p53 levels rebounded. Similarly, in cells expressing 5 or 8E6, p300 levels were much lower than in control cells in the absence if inhibitor; however upon pre-incubation with either MG132 or lactacystin p300 levels increased. Thus, the lower levels of p300 protein seen in 5 and 8E6 expressing cells is mediated by the proteasome. The proteasome inhibitors also caused a slight increase in p300 levels in the LXSN, 38E6 and 16E6 expressing cells, suggesting a general mechanism for regulating p300 stability. It should be noted that although we have demonstrated that the proteasome is involved in the modulation of p300 levels, we can not rule out the possibility that this is occurring through the proteasomal degradation of another factor involved in p300 translation, rather than via direct degradation of p300 itself.

16E6 is known to mediate the proteasomal degradation of many proteins through an interaction with the E3 ubiquitin ligase E6AP [11]. Importantly, E6AP has been shown to interact weakly with many of the β-HPV E6 proteins [30]. We therefore wished to determine if E6AP was involved in HPV 5 and 8E6 mediated degradation of p300. LXSN control and E6 expressing cells were transfected with pools of 4 individual siRNAs directed against E6AP or non-targeting controls. Cells were harvested 72 hr post-transfection and assayed by immunoblot for E6AP, p53, and p300 (Figure 3C). Importantly, in all cells transfected with the pooled siRNAs targeting E6AP, E6AP protein levels were drastically reduced. This was confirmed at the mRNA level using real-time RT-PCR (data not shown). Very low basal levels of E6AP were present in 16E6 cells, consistent with previous observations that E6AP is auto-ubiquitinated and degraded in 16E6-expressing cells [41].

As above, p53 was used as a control, as it is known to be degraded in an E6AP-dependent manner. In 16E6 cells transfected with the pooled control siRNA, p53 levels were decreased as compared to LXSN control cells. Conversely, when these cells were transfected with a pool of siRNAs targeting E6AP, p53 levels increased. Surprisingly, the E6AP targeting siRNA pool had no effect on the levels of p300 in either 5 or 8E6 expressing cells. p300 levels were lower than control cells in the presence of the control siRNA pool, and remained unchanged upon transfection with the E6AP targeting pool, indicating that p300 degradation was E6AP independent in these cells. To verify these results and minimize any potential off target effects from a pool of 4 siRNAs we repeated these experiments using 2 of the 4 siRNAs from the pool individually. Similar to the results seen with the pooled siRNAs, each individual siRNA was able to knockdown the majority of E6AP in the cell (Figure 3D). Likewise, the effects on p53 and p300 were similar to that seen above. In 16E6 expressing cells p53 protein levels rebounded in cells transfected with either targeting siRNA, while the siRNAs had no effect respect to p300 levels in either 5 or 8E6 expressing cells. Taken together these data demonstrate that while p300 degradation is indeed dependent on the proteasome, it is independent of the E3 ubiquitin ligase E6AP.

### p300 degradation is modulated by AKT activity

**Figure 4:**
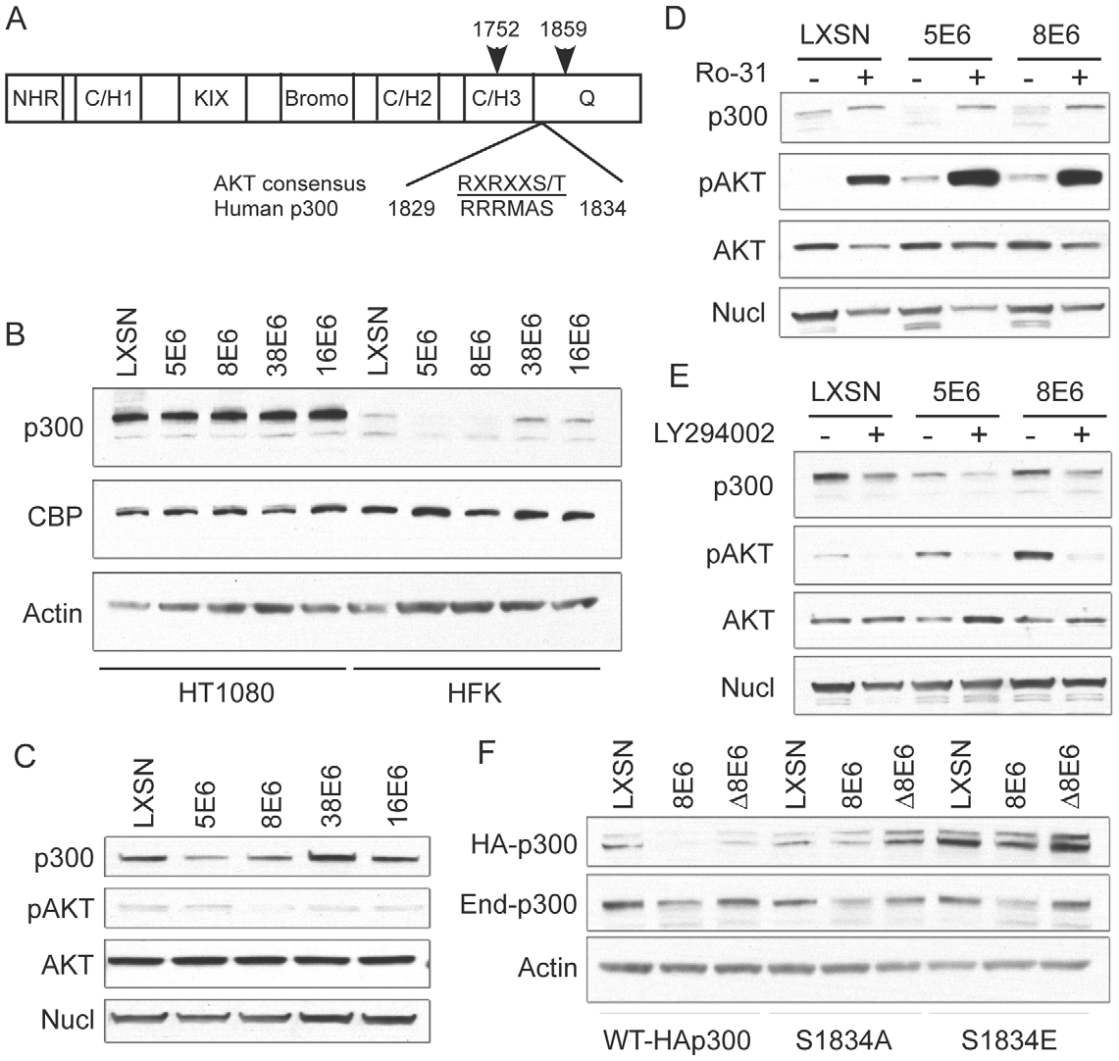
p300 degradation is modulated by AKT activity. (A) Domain structure of p300 demonstrating the location of the AKT phosphorylation site with respect to the C/H3 and Q domains [modified from 44]. Arrows indicate the start and end of the C/EBPβ binding region that is also the binding site of many other proteins including E6 (B) Representative immunoblot showing levels of p300, CBP and nucleolin (control) in LXSN or E6-expressing HT1080s and HFKs. (C) Representative immunoblot showing levels of p300, pAKT, total AKT and Nucleolin in LXSN and E6-expressing cells. (D) LXSN and E6-expressing HFKs were treated with DMS0 (−) or Ro-31(+) 2 hr prior to harvesting cell lysates. Lysates were then analyzed by immunoblot for p300, phospho-AKT, AKT and nucleolin. (E) LXSN and E6-expressing HFKs were treated with DMS0 (−) or LY294002 (+) 24 hr prior to harvesting cell lysates. Lysates were then analyzed by immunoblot for p300, phospho-AKT, AKT and nucleolin. (F) Representative immunoblot showing levels of transiently transfected HA-p300, endogenous p300 and Actin as a loading control in LXSN, 8E6 and Δ8E6 cells transfected with WT-HAp300 or p300 S1834A/E mutants.

As p300 degradation was shown to be proteasome-dependent, but E6AP-independent, we wished to determine the mechanism by which E6 may affect degradation. Importantly, p300 stability has previously been shown to be modulated by AKT activation [42]. A consensus AKT phosphorylation site is located within the p300 C-terminus, between amino acids 1829–1834, near the CH3 and Q regions of the protein [43], [44] (Figure 4A). Phosphorylation of the S1834 within this site has been shown to be required for stabilization of the p300 protein; if this site is mutated or AKT signaling inhibited, then p300 protein is targeted for degradation in a proteasomal-dependent manner. In support of a role for AKT in β-HPV 5 and 8E6 mediated p300 degradation, we found that p300 levels were not lowered in 5- and 8E6 expressing HT1080 cells, which harbor constitutively active AKT [45] (Figure 4B). Importantly, p300 protein levels in these samples were analyzed with an antibody specific only to p300, and not cross-reactive to its related protein, CBP. In contrast, an antibody specific to CBP showed equal CBP protein levels in all samples from both HFK and HT1080. Additionally, there were no differences in AKT or pAKT levels between LXSN control or any of the E6 expressing cells within their respective cell lines (Figure 4C), indicating that E6 expression itself was not perturbing cell-wide AKT activation. Thus, while AKT activation appears to be involved in 5 and 8E6 mediated degradation of p300, AKT activation itself is not altered by E6 expression.

To further demonstrate the importance of AKT in E6 mediated p300 degradation, we employed the use of an activator of AKT signaling, Ro 31-8220 [46], and an inhibitor of AKT signaling, Ly294002 [47]. We hypothesized that activating AKT with Ro 31-8220 would increase p300 levels in 5 and 8E6 expressing cells, as the protein would no longer be targeted for proteasomal degradation. Conversely, inhibiting AKT with Ly294002 would destabilize p300 across all of the cell lines, and total p300 protein levels would be lower than their corresponding non-drug treated controls. Indeed, when cells were pre-treated with Ro 31-8220 to activate AKT, p300 levels were increased in all cells, but most dramatically in 5 and 8E6 expressing cells, which initially harbored the least amount of p300 to begin with (Figure 4D). Conversely, when cells were pre-treated with Ly294002 to inhibit AKT, p300 levels were depleted in both LXSN control, and E6 expressing cells (Figure 4E). The observation that p300 protein levels also change in non-E6 expressing cells is expected as this is a normal mechanism of p300 regulation in the absence of E6. Also of note, the levels of pAKT in the lysates from 5 and 8E6 expressing cells used in Figure 4D and E suggest that 5 and 8E6 may be activating AKT, differing from the data shown in Figure 4C where no such difference was seen. Over most experiments we consistently saw no differences in AKT activation, as stated above.

Finally, utilizing a pCMV-HA-p300 expression vector, we generated two mutants of p300 at the AKT phosphorylation site, S1834. These mutants included S1834A, which should not be able to be phosphorylated by AKT and thus shouldn't be affected by E6 expression; and S1834E, in which the serine is replaced by glutamic acid, and thus represents a phospho-mimic which should also not be affected by E6 expression, and additionally should stabilize the protein leading to higher levels when assessed by immunoblotting. Both of these mutants have been previously described and tested with respect to AKT mediated effects on p300[43]. LXSN, 8E6 and Δ8E6 expressing HFKs were transiently transfected with either WT-HAp300, HAp300-S1834A, or HAp300-S1834E. 48 hours post-transfection, samples were harvested and assessed by western blot with an antibody to HA, endogenous p300, and Actin as a loading control (Figure 4F). WT-HAp300 mimicked the endogenous p300 in that both proteins were present in lower levels in 8E6, but not Δ8E6 expressing cells, when compared to LXSN. HAp300-S1834A and HAp300-S1834E both showed minimal decrease in their respective protein levels in 8E6 expressing cells, as compared to LXSN or Δ8E6 expressing cells, indicating that mutation at this site significantly abrogates 8E6-mediated degradation of p300. Moreover, total levels of HAp300-S1834E were much higher in all three cell lines than that of WT or the S1834A mutant p300, indicating that this protein has been stabilized due to the glutamic acid acting as a phospho-mimic at this site. Finally, all cell lines were re-probed with a p300 antibody to gauge the levels of endogenous p300 protein within each sample. In all cases, regardless of which p300 expression vector was used for transient transfection, endogenous p300 levels were lower in the 8E6 cell lysates as compared to LXSN or Δ8E6 expressing lysates. Taken together, these data further confirm that 5 and 8E6 mediated p300 degradation involves AKT signaling.

### E6 competes with AKT for binding to p300

**Figure 5:**
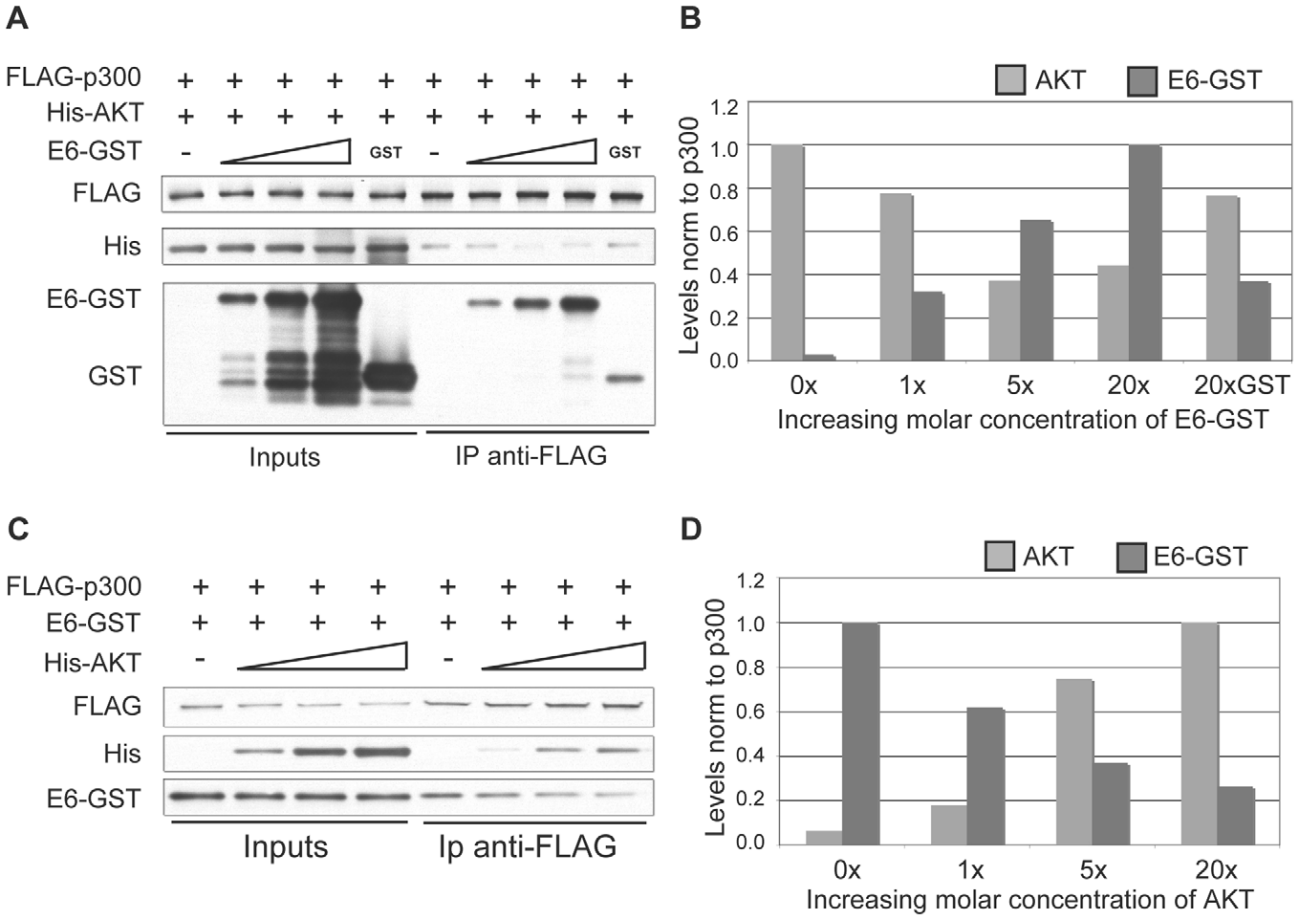
E6 and AKT compete for binding to p300. (A) Representative immunoblot showing the levels of AKT and E6 associated with p300 under increasing molar input of E6. Input protein levels are shown at the left and represent 5% of input protein. (B) Quantitation of E6 and AKT levels from A. 20X molar GST is shown as a control. (C) Representative immunoblot showing the levels of AKT and E6 associated with p300 under increasing molar input of AKT. Input protein levels are shown at the left and represent 5% of input protein. (D) Quantitation of E6 and AKT levels from C.

The AKT phosphorylation site in the p300 C-terminus lies within a well-defined region of p300 that has been shown to be the binding site of numerous other p300-interacting proteins, including E6. 8E6 interaction with p300 at this region has been finely mapped, and has been shown to bind to the region encompassing aa 1770–1814 [33], which is directly adjacent to the AKT phosphorylation site at aa 1829–1834. We hypothesized that due to the proximity of the AKT and E6 binding sites on p300, strong binding of 8E6 would in turn prevent AKT from binding, phosphorylating and in turn stabilizing p300, thus leading to p300 degradation. To test this hypothesis a competitive binding assay was performed using purified GST-tagged 8E6, FLAG-tagged p300 and His-tagged AKT. Beginning with equimolar amounts of p300 and AKT, increasing molar amounts of 8E6 were introduced. The complexes were then pulled down using anti-FLAG agarose beads, and eluted samples subjected to SDS-PAGE and immunoblot analysis for each of the proteins (Figure 5A and B). As can be seen, as greater amounts of E6 was added, more E6 was pulled down with p300, and correspondingly less AKT was recovered. The experiment was then repeated by starting with equimolar amounts of p300 and 8E6, followed by the addition of increasing molar amounts of AKT. Once again, complexes were pulled out using anti-FLAG agarose beads and analyzed as above (Figure 5C and D). Similarly, as greater amounts of AKT were added, more AKT was found to be associated with p300, and correspondingly less E6 was recovered. Taken together, these results demonstrate that E6 and AKT compete for binding to p300, and imply that this competition could preclude AKT from phosphorylating the activation site within the p300 C-terminus, thus leading to de-stabilization of p300 protein levels.

### 5 and 8E6-mediated p300 degradation modulates the levels of multiple markers of keratinocyte differentiation

**Figure 6:**
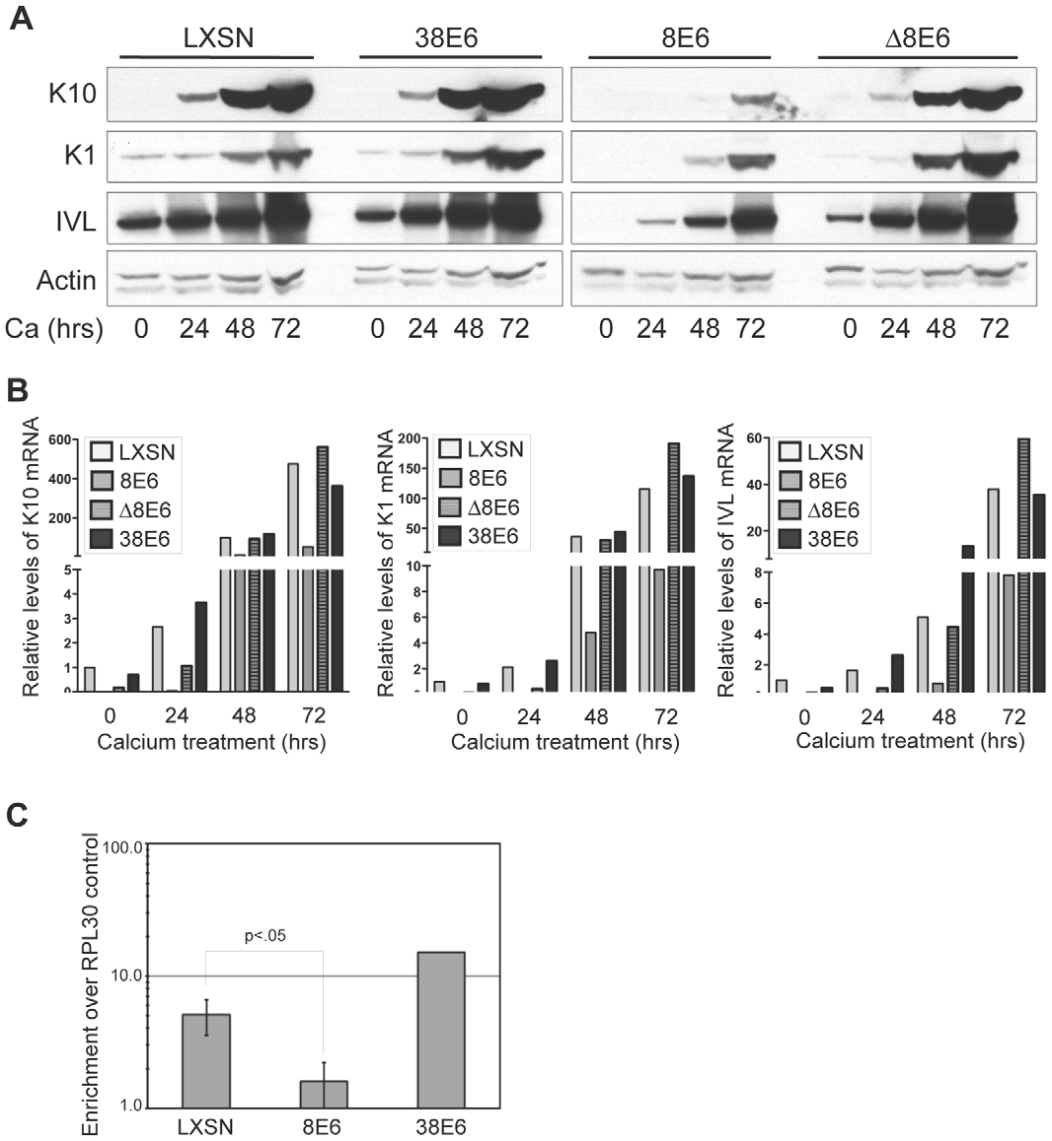
p300 degradation alters K1, K10 and IVL expression. (A) Representative immunoblot showing levels of K1, K10 and IVL protein in LXSN, 38E6, 8E6 and Δ8E6-expressing HFKs during 72 hr calcium-induced differentiation. Actin levels are shown as a loading control. (B) K1, K10 and IVL mRNA levels levels in LXSN 38E6, 8E6 and Δ8E6-expressing HFKs during 72 hr calcium-induced differentiation. Relative levels of each mRNA were calculated using the ΔΔCT method with GAPDH to normalize mRNA levels within each sample. Values shown are fold-change in each sample compared to the LXSN (0 hr) vector control. (C) ChIP analysis using anti-p300 antibodies to pull down chromatin from LXSN control, 8E6 and 38E6-expressing HFKs, with quantitation by real-time PCR. Values represent the enrichment of p300 at the IVL promoter shown relative to the IgG and RPL30 negative controls. Error bars represent standard deviation (n = 3, p<.01 by 2-tailed student's T-test).

Reduced levels of p300 protein in 5 and 8E6-expressing cells has the potential to affect a multitude of signaling pathways. One such pathway that has recently been shown to be specifically abrogated by p300 knockdown is keratinocyte differentiation [48]. Introduction of shRNAs targeting p300 has been shown to attenuate both an early differentiation marker, K1 and a late differentiation marker, filaggrin, in both calcium-induced and raft-culture model systems of keratinocyte differentiation [48]. Additionally, the expression of another differentiation marker, involucrin, has previously been demonstrated to be regulated by p300 at the transcriptional level [49], [50]. We examined the protein levels of three differentiation markers; K10, K1 and involucrin in both untreated, and calcium-differentiated cells harboring either 8E6, Δ8E6, 38E6 or LXSN vector (Figure 6A). As expected, as calcium-induced differentiation was allowed to proceed (see Figure S1 for AKT/pAKT levels during differentiation, and Figure S2 for cell morphology during differentiation), the levels of K1, K10 and involucrin all increased substantially in LXSN control cells. Importantly however, while a slight increase of each protein was observed in 8E6-expressing cells over the timecourse of differentiation, the levels of K1, K10 and involucrin in 8E6 cells relative to that in control LXSN cells was dramatically lower; not only in untreated cultures, but also during each timepoint during differentiation. Strikingly, this abrogation of differentiation was not seen in 38E6 expressing cells, indicating that this effect is not simply a general response to HPV-E6 expression. Moreover, the attenuated expression of differentiation markers was also not seen in Δ8E6-expressing cells, indicating that p300 binding and/or degradation are necessary to achieve this effect. To determine if attenuation of differentiation marker expression was at the level of transcription or translation, we examined the same cultures for mRNA expression using real-time RT-PCR (Figure 6B). As seen with the levels of protein expression, the levels of K1, K10 and involucrin mRNA all increased in LXSN control cells following the induction of calcium-mediated differentiation. Likewise, while a slight increase in the expression of all three genes was discernable in 8E6 expressing cells, the relative levels of each gene was drastically reduced by 8E6 expression when compared to the corresponding LXSN control sample. Finally, as seen with the protein levels, both this attenuation of expression was almost completely absent in both 38E6 and Δ8E6-expressing HFKs. Thus, 8E6 expression attenuates the mRNA levels of three different markers of differentiation; K1, K10 and involucrin.

As p300 has been shown to associate with the involucrin promoter and enhance its transcription [49], [50], we hypothesized that p300 occupancy at the involucrin promoter would be attenuated in 8E6 expressing cells, as these cells harbor significantly less p300 protein. We performed ChIP analysis using antibodies to p300 and primers to amplify the region of the involucrin promoter known to be bound by p300 (Figure 6C). Using occupancy at the RPL30 promoter (control provided in the ChIP kit), and binding of IgG as controls, we verified that p300 is approximately 6-fold enriched at the involucrin promoter in LXSN cells, and over 10-fold enriched at the involucrin promoter in 38E6 cells. Conversely, p300 was almost completely absent from the involucrin promoter in 8E6 cells.

**Figure 7:**
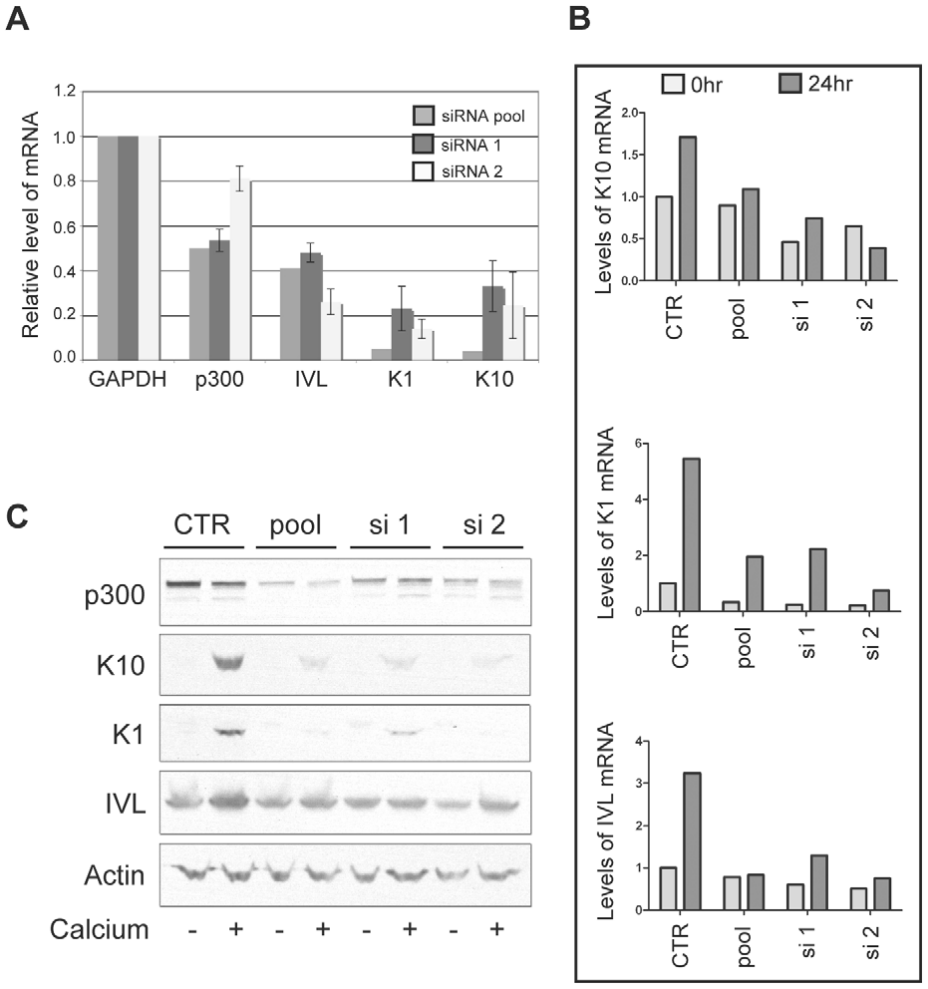
p300 knockdown by siRNA is sufficient to attenuate the mRNA and protein expression of K1, K10 and involucrin. (A) p300 was knocked down using either a pool of 4 siRNAs (pool) or 2 individual siRNAs (si 1 and si 2) and compared to cells mock treated with a non-targeting siRNA (CTR). 72 hr post transfection, lysates were harvested and analyzed for p300, K1, K10 and IVL mRNA levels. Relative levels of each mRNA were calculated using the ΔΔCT method with GAPDH to normalize mRNA levels within each sample. Values shown are the fold-change in each sample compared to the siRNA control. Error bars represent standard deviation (n = 2). (B) p300 was knocked down using either a pool of 4 siRNAs (pool) or 2 individual siRNAs (si 1 and si 2) and compared to cells mock treated with a non-targeting siRNA (CTR). 48 hr post transfection, cells were treated with either normal media or media containing 1.5 mM CaCl_2_ to induce differentiation. 24 hr later lysates were harvested and analyzed for K1, K10 and IVL mRNA levels. Relative levels of each mRNA were calculated using the ΔΔCT method with GAPDH to normalize mRNA levels within each sample. (C) A parallel set of samples was treated as in B, and 24 hr later lysates were harvested and analyzed by immunoblot for p300, K1, K10, IVL and Actin (control).

While p300 has been shown to play a role in the transcriptional regulation of involucrin [49], [50], a direct role for the involvement of p300 in the transcriptional regulation of K1 and K10 has not been described. Therefore, to more thoroughly demonstrate that p300 degradation by itself is directly responsible for the altered expression of each of the differentiation marker examined in Figure 6, we employed the use of siRNA pools and two individual siRNAs to knockdown p300 in non-E6 expressing cells, and subsequently examine both the mRNA and protein from the resulting cell lysates. In undifferentiated cell lysates, knockdown of p300 by either an siRNA pool or two individual siRNAs resulted in lower relative levels of K1, K10 and involucrin mRNA (Figure 7A). When extended to a calcium-differentiation model, this trend was maintained following 24hr treatment with calcium-media; while the mRNA (Figure 7B) and protein (Figure 7C) levels for each gene increased dramatically during calcium treatment in control cells, levels of K1, K10 or involucrin mRNA and protein either stayed the same or increased only slightly in cells transfected with p300 siRNA. The inability to see dramatically lower levels of K1, K10 or involucrin mRNA or protein in undifferentiated cells upon knockdown of p300 (Figure 7B and C) could be due to an inability to detect a difference in samples treated with siRNA for only 48 hrs (as opposed to the 72 hrs used for Figure 7A), or may be due to a dose-effect from the level of p300 knockdown itself in this particular experiment. Under more standard siRNA conditions however (as used for Figure 7A), decreased mRNA expression of each gene is consistently reproducible. Additionally, the effect of p300 siRNA on IVL protein expression in Figure 7C is not as dramatic as that seen with 8E6 expression in Figure 6A. As above, this may be due to inadequate knockdown of p300 at this particular time, or it may indicate that additional functions of 8E6 other than p300 degradation may affect IVL levels. Taken together, these data demonstrate that 8E6-expressing cells are attenuated in their ability to undergo calcium-mediated differentiation, and this attenuation is directly mediated by the decreased levels of p300.

## Discussion

In order to identify host-cell proteins that interact with β-HPV E6, we performed GST-pulldowns using GST-tagged E6 proteins and whole cell HFK lysates, followed by mass spectrometry analysis of the interacting complexes. We identified p300 as a protein that interacted with all of the E6 proteins tested; albeit with different relative strengths and different consequences to downstream signaling. While p300 binding to 16E6, 8E6 and most recently 38E6, has previously been demonstrated [33], [34], [36], [37], the relative strengths of the two interactions had not been examined, and the consequences of 8E6-p300 binding had not been studied with respect to effects on host cell signaling. Here we demonstrate not only that other β-HPV E6 proteins are also able to interact with p300, but also show that β-HPV 5 and 8E6 bind to p300 at relatively high levels, while β-HPV 38E6 and α-HPV 16E6 bind to the protein at much lower levels.

The importance of p300 for a myriad of signaling and regulatory pathways has been well documented. The protein plays a role in bridging other transcription factors to the basal transcriptional machinery; acetylating histones to facilitate chromatin remodeling; regulating DNA repair, cell growth, differentiation and cell death; is involved in embryogenesis; and functions as a tumor suppressor [Reviewed in 39,40]. Not surprisingly, p300 has been shown to be mutated in a number of cancers, and targeted by many viruses. p300 has been shown to be inactivated by C-terminal truncation in a small percentage of cancers of epithelial origin, including colorectal, gastric, breast, pancreatic, cervical and ovarian, as well as in the human diffuse B-cell lymphoma cell line RC-K8 [51], [52], [53], [54], [55], [56]. Mutations in p300 are even more common in colorectal cell lines, where 4/17 were found to harbor homozygous or heterozygous mutations [54]. Moreover, ectopically expressing p300 in cancer lines harboring biallelic mutations of p300 slows cell growth [57], and p300 knockout mice have been shown to develop histiocytic sarcomas [58]. Viral inactivation of p300 has been shown to be mediated by oncoproteins from five different viruses; adenovirus E1A, SV40 large T antigen, E6 and E7 from HPV, Tax protein from HTLV-1 and A238L protein from African Swine Fever Virus [59], [60], [61], [62], [63], [64], [65]. Importantly all of these oncoprotein/p300 interactions interfere with the acetyltransferase and/or transactivation ability of p300, which in turn mediates tumorigenicity. Interestingly, recent data has shown that the promoters for p300 regulated genes vary greatly with respect to their affinities for p300 [66], thus lowering levels of p300 (even slightly) may have drastic consequences for the expression of genes with a relatively low affinity site for p300, and possibly no effect on genes/promoters with high affinity sites. Thus, even slight alterations of this pathway by viruses or mutation can have profound effects on cellular signaling.

Importantly, we demonstrate that the strong binding observed between p300 and either β-HPV 5 or 8E6 does indeed have a functional consequence for the host-cell, as the interaction inhibits the association of AKT with its normal binding site at the p300 C-terminus, thus targeting the p300 protein for proteasomal degradation. We provide additional evidence that altering the levels of E6 expression via siRNA knockdown reverses this affect in somewhat of a dose-response dependent manner, suggesting that differences in 5 or 8E6 expression may alter the degree of p300 degradation observed. This is important because it is still controversial as to what the physiological levels of β-HPV E6 proteins may be in HPV infected individuals. Regardless, the general effects of p300 knockdown in both primary and immortal cell lines has been well studied. In primary HFKs, p300 knockdown with shRNA has been shown to delay differentiation, allow differentiated HFKs to re-enter the cell-cycle, increase cell proliferative capacity, extend the lifespan of cells in culture, and regulate the acetylation and expression of p53 [48]. Additionally, cells lacking p300 have been shown to exhibit changes characteristic of epithelial to mesenchyme transition, including gene expression changes, loss of cell-cell adhesion, defects in cell-matrix adhesion and increased migration through collagen and matrigel [67]. With respect to cell death, p300 has been shown to regulate the sensitivity of cells to irradiation, and has a pro-apoptotic function in the DNA damage response. Thus mutations in tumor cells that attenuate p300 function confer resistance to ionizing radiation and other genotoxic agents [68]. Finally, in the context of 16E6, p300 knockdown by shRNA was shown to induce transcription of hTERT mRNA and induce telomerase activity [69]. Thus the discovery of p300 degradation in HPV 5 and 8 expressing cells has the potential to have far-reaching consequences.

Indeed, our finding that the decreased p300 levels in 8E6 expressing cells leads to the attenuated expression of multiple structural markers of differentiation has profound implications for the HPV replicative cycle, as the ability of HPV to replicate depends on continued cell proliferation and inhibition of terminal differentiation. Recent studies have focused on K10 as a possible tumor suppressor, as overexpression of this particular cytokeratin has been shown to both inhibit cell proliferation and suppress tumor development [70], [71], [72]. Thus, by inhibiting the expression of K10 via p300 degradation, 8E6 functions to remove this blockade. Moreover, in a transgenic mouse model, K1 and K10 expression were shown to be lost during skin tumor progression [70]. Interestingly, loss of K1 expression and altered K10 localization have been previously demonstrated both in cultured cells expressing the HPV 8 early region [73], and from papillomous lesions taken from EV patients [74], however the mechanisms by which these events occurred were not known. Importantly, our data has now identified a link between E6 expression, p300 degradation and decreased expression of important differentiation markers within keratinocytes.

Another important function of p300 that is of particular relevance to HPV infection is that of regulating the activity of p53, and as a co-factor in p53-dependent transactivation of a number of genes. Following DNA damage, p300 acetylates p53 on Lys 382, which stabilizes the p53/DNA complex at target promoters [75], [76], [77]. Additionally, p300 can be recruited by p53 to certain promoters, where it can act as a bridge for other transcription factors, or act to acetylate histones [78], [79]. Importantly, E6 from both high-risk and low-risk α-HPVs, as well as BPV-1 and most recently β-HPV 38E6 have been shown to inhibit the ability of p300 to transactivate p53 [34], [35], [36], [37], [38]. This function of E6 is independent of E6AP and does not require p53 degradation. Rather it is thought that the interaction between E6 and p300 may inhibit the association of p53 with p300 [34]. Thus, while only high-risk α-HPV E6 proteins are able to disrupt p53 signaling by degrading the p53 protein itself, E6 from low-risk α-HPVs are capable of interfering with this pathway simply through association with p300. It is therefore tempting to speculate that the degradation of p300 by β-HPV 5 and 8E6 represents yet another way in which this pathway may be disrupted by the E6 oncoprotein. Preliminary evidence from our lab suggests that HPV 5, 8 and 38E6 are all capable of attenuating p300-mediated acetylation of p53, and further studies are underway to examine other effects these oncoproteins may have on p53 signaling.

In summary, we have identified p300 as a protein that appears to broadly interact with the E6 oncoprotein from both alpha and beta genera of HPV. Importantly, due to the observed differences in the strength of this association among HPV types, different consequences to p300 signaling occur. The relatively weak association with 16E6 has previously been shown to allow for inhibition of p53 signaling [34], [36], [38], however does not perturb the steady state levels of p300 protein within the cell. Conversely, 5 and 8E6 bind to p300 at relatively high levels, causing the stability of the p300 protein becomes perturbed, leading to an overall decrease of the p300 levels within the cell. As a result, certain proteins that depend on p300 for their regulation, like K1, K10 and IVL become de-regulated, altering their expression level, and in turn leading to additional consequences with regard to downstream signaling events. Importantly, given the broad role that p300 plays in orchestrating the enhancement or repression of transcriptional processes within the cell, the consequences of p300 degradation are likely to have far-reaching effects with regard to HPV infection.

## Materials and Methods

### Reagents

Antibodies to GST(B-14), Nucleolin(C-23), E6AP(H-182), His(H-15), EGR-1(588), p300(N-15) Actin (I-19), Cytokeratin 10/13 (DE-K13) and Involucrin (SY8) were purchased from Santa Cruz Biotechnology; antibodies to AKT, phospho AKT (Ser 473) and CBP were purchased from Cell Signaling Technology; antibodies to HA (HA.11) and Keratin 1 were purchased from Covance; antibodies to p300 (NM11) were purchased from BD Pharmingen; antibodies to p53(DO-1) were purchased from Calbiochem; non-cross reactive p300 antibodies (RW128) were purchased from Millpore. siRNA pools and individual siRNAs specific for E6AP, p300 and controls were purchased from Dharmacon RNA Technologies. siRNA's designed to target 5E6 and 8E6 were designed and purchased from Invitrogen (see Table S3 for sequences). Proteasome inhibitors Lactacystin and MG132 were purchased from Calbiochem and used at a final concentration of 20 µM. AKT inhibitor LY294002 was purchased from Cell Signaling Technology and used at a final concentration of 10 µM. AKT activator Ro 31-8220 was purchased from Sigma and used at a final concentration of 10 µM. Purified recombinant FLAG-p300 and recombinant His-AKT1 were purchased from Active Motif. EZ-view Red anti-FLAG M2 affinity gel was purchased from Sigma.

### DNA constructs and mutagenesis

All HA-tagged and untagged E6 constructs have been described previously [29], [30], with the exception of Δ8E6, which was constructed by performing site-directed mutagenesis to obtain a deletion of aa 132–136 [33] (see Table S3 for mutagenesis primers). Expression of E6 was verified at the mRNA level following selection (see below) by real-time RT-PCR analysis. All primers have been previously described [29]. New primers were designed to assess levels of Δ8E6 (see Table S3), as the original 8E6 primers flanked the deletion site. HA-p300-pCMV was purchased from Upstate Biotech, and used to construct S1834A/E mutants via site directed mutagenesis (see Table S3 for mutagenesis primers).

### Tissue culture

Primary human foreskin keratinocytes (HFKs) were derived from neonatal human foreskins and grown in EpiLife medium supplemented with calcium chloride (60 µM), human keratinocyte growth supplement (Cascade Biologics, Portland, OR) and penicillin-streptomycin. 293T cells were grown in Dulbecco's modified Eagle's medium (Gibco-BRL) containing 10% fetal bovine serum (FBS) and penicillin-streptomycin. For calcium-induced differentiation, confluent monolayers of HFKs were treated by withdrawal of growth factors and addition of media containing 1.5 mM CaCl_2_. Stable E6-expressing cell lines were produced using transient vesicular stomatitis virus G (VSV-G) pseudo typed virus as previously described [29]. Briefly, E6 proteins cloned into an LXSN vector were co-transfected with VSV-G helper plasmids into 293T cells using Fugene 6 (Roche), and retrovirus collected at 12, 24, 36 and 48 hrs post transfection. Transiently produced virus was concentrated by ultracentrifugation and used to infect HFK monolayers (50 to 60% confluent) in the presence of Polybrene (8 µg/mL). Four hours after infection, cells were washed with PBS and the media replaced. The cells were expanded when confluent and were placed under neomycin-G418 selection (50 mg/liter) for 7 days. HA-p300WT, HA-p300S1834A and HA-p300S1834E were all transiently transfected into LXSN, 8E6 and Δ8E6 cells using TransIT Keratinocyte transfection reagent (Mirus), and assessed 72 hr later for levels of both endogenous p300 and the transfected HA-p300 construct.

### GST-protein purification and GST pull-down assays

Generation of N-terminally GST tagged E6 vectors was described previously [29]. GST-E6 constructs were transformed into BL21-AI *Escherichia coli*, and grown overnight at 37°C on LB plates containing 50 µg/ml ampicillin. Isolated colonies were used to inoculate 20 ml LB broth containing 200 µg/ml carbenicillin and grown overnight with shaking at 37°C. 10 ml of the overnight culture was added to 1 L of fresh LB-carbenicillin and incubated at 37°C with shaking for 2.5 hrs. Cultures were then transferred to room temperature and incubated for 30 min with shaking, after which the optical density at 600 nm of all cultures was between 0.4 and 0.6. L-Arabinose was added to each culture at a final concentration of 0.2% to induce protein expression, followed by growth for 4 hr at room temperature with shaking. Bacterial cells were then harvested by centrifugation at 6,000 rpm for 15 min at 4°C, and the resulting pellets were stored at −20°C. Bacterial pellets were resuspended in PBS-P (phosphate-buffered saline, 50 mM EDTA and protease inhibitor tablets) and lysed via two passages through a microfluidizer, followed by a 30 min incubation with 0.1%TritonX-100 at 4°C with end-over-end rotation. Bacterial lysates were centrifuged at 14,000 rpm in a JA17 rotor for 15 min at 4°C, and the pellets discarded. The resulting supernatants were added to pre-equilibrated glutathione-sepharose bead slurries, and incubated at 4°C for 1 hr with end-over-end rotation. The bead slurries were then washed four times with PBS-P, followed by elution of the bound GST proteins with 20 mM GSH/50 mM Tris-CL for 1 hr at 4°C with end-over-end rotation. Eluted proteins were collected by centrifugation of the bead slurry, and aspiration of the protein-containing supernatant. A total of two elutions were carried out in this manner. The two elutions for each GST-E6 protein were then combined, and dialyzed using Zeba Desalt Spin Columns (Pierce, Rockford, IL) and protein buffer (5 mM Tris, 100 mM KCl, 0.5 mM EDTA, 1 mM DTT, 5% glycerol, 0.1%NP40, and protease inhibitor tablets).

For GST pulldown assays, equal amounts of GST-tagged proteins were incubated with pre-cleared whole cell HFK lysates in dialysis buffer (5 mM Tris-HCL pH 7.4, 100 mM KCL, 0.5 mM EDTA, 1 mM DTT, 5% glycerol, 0.1% NP-40, and protease inhibitor tablets) and gently agitated for one hour at 4°C. Glutathione sepharose 4B beads were added to each pulldown, incubated at 4°C for two hours, washed in binding buffer, and recovered by boiling in 2X sample buffer. The samples were separated on SDS-polyacrylamide gels, and analyzed by immunoblot or for mass spectrometry at our proteomics facility (FHCRC).

### Co-Immunoprecipitation

HA-tagged E6 expressing HFKs were harvested in NP-40 lysis buffer (1x PBS, 0.5% NP-40, 10% glycerol, 10 µM zinc chloride, 2 mM dithiothreitol, 80 mM β-glycerophosphate, 50 mM sodium fluoride, 1 mM sodium orthovanadate, and a COMPLETE protease inhibitor tablet [Roche, Alameda, CA]). Cells were lysed by sonication for 1 min at 50% duty. Cell debris was pelleted at 14,000 rpm for 15 min and lysates were precleared by rotating at 4°C with 50 µL of protein G agarose (Roche, Alameda, CA). After centrifugation to remove the beads, lysates were incubated with the appropriate antibody for 1–2 h at 4°C and purified by adding protein G agarose and rotating for another hour at 4°C. Immunocomplexes were washed three times with lysis buffer and eluted by heating for 10 min at 70°C in 2x sample buffer. Elutions were electrophoresed on NuPAGE 4%–12% Tris-Bis gradient gels (Invitrogen, Carlsbad, CA) to resolve HA-tagged E6 proteins and immunoblotted for p300 or HA as described.

### RT-PCR

RNA was isolated with Trizol reagent (Invitrogen, Carlsbad, CA) as previously described [29]. 1 µg of total RNA was reverse transcribed to generate cDNA, using the iScript cDNA synthesis kit (BioRad, Hercules, CA). As a negative control, parallel samples were run without reverse transcriptase. Non-quantitative PCR amplification was then performed to identify 100 bp amplicons with E6 and 36B4 primers as previously described [29]. For real-time RT-PCR, RNA was isolated and reverse transcribed as above, and quantitative real-time PCR was performed using an ABI 9700 sequence detection system (Applied Biosystems, Foster City, CA). Amplification was carried out using TaqMan master mix and the following pre-designed Taqman primer/probes: GAPDH (4333764F), p300 (Hs00914223_m1), Krt1 (Hs00196158_m1), Krt10 (Hs00166289) and IVL (Hs00846307_s1) according to the manufacturer's instructions (Applied Biosystems, Foster City, CA). Reactions were performed in triplicate in a 25 µl volume, with the following cycle parameters: enzyme activation (10 min at 95°C), followed by 40 cycles (each cycle consisting of 15 seconds at 95°C and 1 in at 60°C). Data analysis was performed using the comparative threshold cycle method (Applied Biosystems, Foster City, CA) to determine relative expression levels.

### Immunoblotting

Whole-cell lysates were prepared by mechanically detaching cells in cold PBS and resuspending in WE16th lysis buffer (50 mM Tris-HCL at pH 7.5, 250 mM NaCl, 5 mM EDTA, 1% NP-40, 0.1% sodium dodecyl sulfate, 20% glycerol, 80 mM β-glycerophosphate, 50 mM sodium fluoride, 1 mM sodium orthovanadate, and a COMPLETE protease inhibitor tablet [Roche, Alameda, CA]). Lysates were then sonicated and clarified by centrifugation. The DC protein assay (Biorad, Hercules, CA) was used to determine protein concentrations. For immunoblotting of differentiation markers K1, K10 and involucrin, cells were lysed directly in 2X sample buffer (100 mM Tris pH 6.8, 4% SDS, 20% glycerol, 0.8% bromophenol blue). Equal amounts of protein lysates (15 to 30 µg) were electrophoresed on SDS-polyacrylamide gels and transferred to Immobilon-P membranes (Millipore, Billerica, MA). For quantification of western blot data, the membranes were scanned and bands were analyzed by densitometry using ImageJ (NIH).

### Competition assays

*In-vitro* competition assays were performed using a protocol modified from [80]. Briefly, 250 ng of recombinant FLAG-p300 was pre-incubated with equimolar amounts of either His-AKT1 or GST-E6 in modified HAT buffer (50 mM Tris-HCL, pH 8.0, 10% glycerol, 1 mM DTT, 0.1 mM EDTA, 100 mM KCL, 0.1% NP40, Complete protease inhibitor tablet (Roche)) for 1 hr at 4°C with rotation. Increasing concentrations (molar excess 5x–20x), of GST-E6 or His-AKT1 (5x–10x), were added, and further incubated for 1 hr before the addition of 50 µl of anti-FLAG M2 affinity gel. Samples were then rotated at 4°C for 2 hr, washed 4 times with modified HAT buffer, and eluted with 2x SDS-PAGE sample buffer for 5 min at 100°C.

### Chromatin IP

Chromatin immunoprecipitations were performed using the Enzymatic Chromatin IP (Magnetic bead) kit (Cell Signaling Technology), as per the manufacturer's instructions, with minor modifications. Briefly, chromatin from fixed cells was digested to a size range of 150–1000 bases with micrococcal nuclease, followed by brief sonication to disrupt the nuclear membrane. Solubilized chromatin was immunoprecipitated with antibodies to p300(N-15) or IgG control. Antibody-chromatin complexes were pulled-down using ChIP-grade protein-G magnetic beads, washed and then eluted. After cross-link reversal and proteinase K treatment, immunoprecipitated DNA was extracted with phenol-chloroform, and ethanol precipitated. Real-time RT-PCR was performed using SYBR green and primers to the distal AP1 site of the involucrin promoter with the following sequences: 5′-GCTCACACATACCATCTTCTCCTTA-3′ (forward) and 5′-CACCGGTCTTATGGGTTAGCA-3′ (reverse). Standard curves were calculated using serial dilutions of the input sample, and used to calculate the relative amount of product amplified in each reaction. Results were calculated based on the relative enrichment of protein over that seen with the RPL30 control.

### Statistics

All statistics calculations were performed using a two-tailed student's T-test.

## Supporting Information

Figure S1 **Levels of AKT and pAKT during differentiation.** Representative immunoblot showing levels pAKT, and total AKT protein in LXSN, 8E6 and 38E6-expressing HFKs during 48hr calcium-induced differentiation. Actin levels are shown as a loading control.
(TIFF)

Figure S2 **Cell morphology during calcium differentiation.** Representative micrographs of each cell line during a typical calcium differentiation timecourse. All images were acquired immediately prior to sample harvesting at the respective timepoint.
(TIFF)

Table S1 **Proteins identified as potential E6-interactors via mass-spec.** Values represent unique peptides mapping to each protein in each run. Proteins were identified as a potential candidate based on the following filters: DiffScore> = 0.1, Ion percent> = 0.3, and at least 2 unique peptides for a given protein.
(XLSX)

Table S2 **HPV E6 alignment.** Sequence comparisons of HPV E6 proteins at the previously identified 8E6-p300 binding site.
(DOCX)

Table S3 **Primers and sequences.** Sequences of oligos used for siRNA silencing, mutagenesis and RT-PCR.
(DOCX)
