# Supplementary figures and images for: Beta-HPV 5 and 8 E6 Promote p300 Degradation by Blocking AKT/p300 Association
Source: PLoS Pathog. 2011 Aug 25;7(8):e1002211. doi: 10.1371/journal.ppat.1002211 (PMC3161984; doi:10.1371/journal.ppat.1002211)

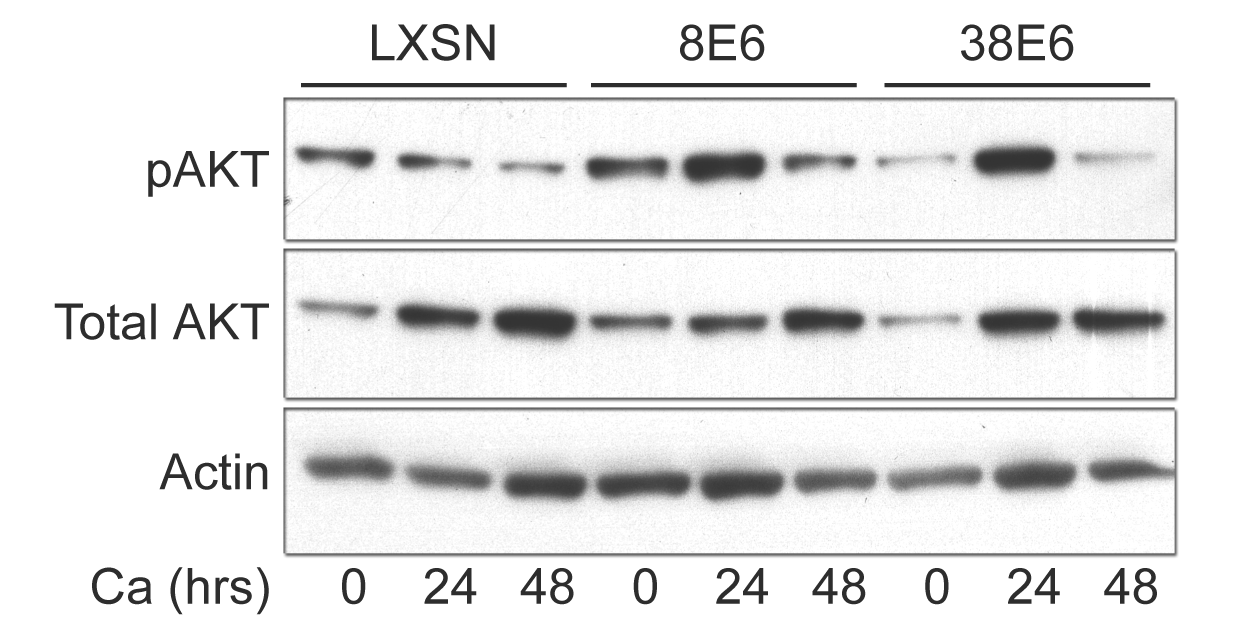

Supplement: Figure S1 — Levels of AKT and pAKT during differentiation. Representative immunoblot showing levels pAKT, and total AKT protein in LXSN, 8E6 and 38E6-expressing HFKs during 48hr calcium-induced differentiation. Actin levels are shown as a loading control. (TIFF) [file ppat.1002211.s001.tiff]

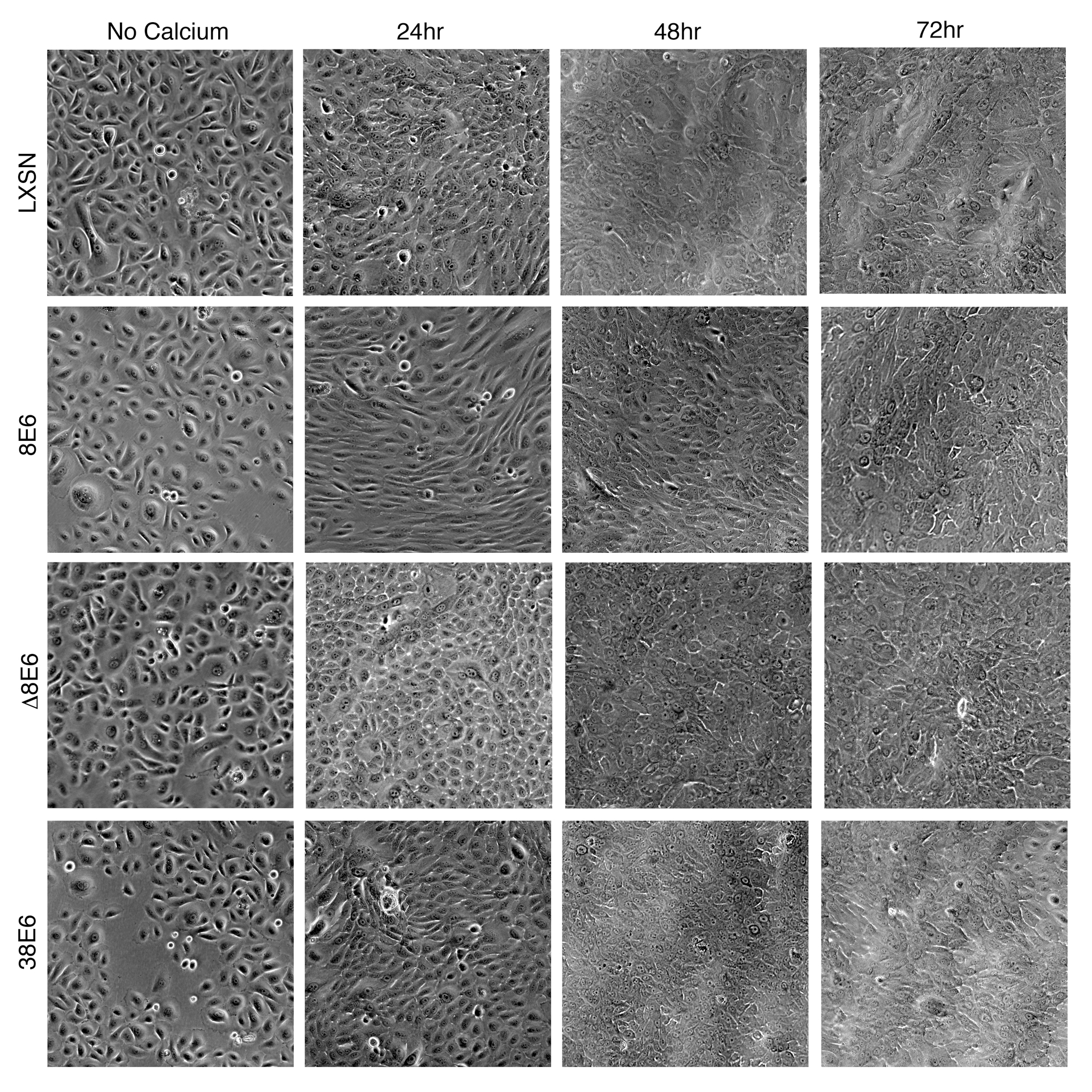

Supplement: Figure S2 — Cell morphology during calcium differentiation. Representative micrographs of each cell line during a typical calcium differentiation timecourse. All images were acquired immediately prior to sample harvesting at the respective timepoint. (TIFF) [file ppat.1002211.s002.tiff]
