# Supplementary material for: Beta-HPV 5 and 8 E6 Promote p300 Degradation by Blocking AKT/p300 Association
Source: PLoS Pathog. 2011 Aug 25;7(8):e1002211. doi: 10.1371/journal.ppat.1002211 (PMC3161984; doi:10.1371/journal.ppat.1002211)
Supplement: Table S2 — HPV E6 alignment. Sequence comparisons of HPV E6 proteins at the previously identified 8E6-p300 binding site. (DOCX) [file ppat.1002211.s004.docx]

| HPV Type | Amino-acid Position | Sequence |
| --- | --- | --- |
| 5E6 | 134-138 | LPFHK |
| 8E6 | 132-136 | RPFHK |
| 38E6 | 121-125 | QAFHK |
| 16E6 | 130-134 | QRFHN |
