# Supplementary material for: Beta-HPV 5 and 8 E6 Promote p300 Degradation by Blocking AKT/p300 Association
Source: PLoS Pathog. 2011 Aug 25;7(8):e1002211. doi: 10.1371/journal.ppat.1002211 (PMC3161984; doi:10.1371/journal.ppat.1002211)
Supplement: Table S3 — Primers and sequences. Sequences of oligos used for siRNA silencing, mutagenesis and RT-PCR. (DOCX) [file ppat.1002211.s005.docx]

| **Name** | **Use** | **Sequence** |
| --- | --- | --- |
| 5E6 siRNA #1 | siRNA silencing | 5’-AGGCAUCCCUGUGAUUGAUUGUUUA-3’ (sense) |
| 5E6 siRNA #1 | siRNA silencing | 5’-UAAACAAUCAAUCACAGGGAUGCCU-3’ (anti) |
| 5E6 siRNA #2 | siRNA silencing | 5’-GGCGCCACUGCAACUUAUGAAUUUA-3’ (sense) |
| 5E6 siRNA #2 | siRNA silencing | 5’-UAAAUUCAUAAGUUGCAGUGGCGCC-3’ (anti) |
| 8E6 siRNA #1 | siRNA silencing | 5’-UAAGGACGAGCUACCCUCUACUAUU-3’ (sense) |
| 8E6 siRNA #1 | siRNA silencing | 5’-AAUAGUAGAGGGUAGCUCGUCCUUA-3’ (anti) |
| 8E6 siRNA #2 | siRNA silencing | 5’-UUGUCAAACUCACACAGUUCUAAGA-3’ (sense) |
| 8E6 siRNA #2 | siRNA silencing | 5’-UCUUAGAACUGUGUGAGUUUGACAA-3’ (anti) |
| Δ8E6 mut F | mutagenesis | 5’-ATTGCTGTGGGAGAGGCGTTAGAGGAGGCTGGAAAGGA-3’ |
| Δ8E6 mut R | mutagenesis | 5’-TCCTTTCCAGCCTCCTCTAACGCCTCTCCCACAGCAAT-3’ |
| p300 S1834A-F | mutagenesis | 5’-CAGGAGGATGGCCGCCATGCAGCGGACTG-3’ |
| p300 S1834A-R | mutagenesis | 5’-CAGTCCGCTGCATGGCGGCCATCCTGGTG-3’ |
| p300 S1834E-F | mutagenesis | 5’-CAGGAGGATGGCCGAGATGCAGCGGACTG-3’ |
| p300 S1834E-R | mutagenesis | 5’-CAGTCCGCTGCATCTCGGCCATCCTGGTG-3’ |
| Δ8E6 qPCR-F | RT-PCR | 5’-AGCTGCGGCTTTAGGTATTC-3’ |
| Δ8E6 qPCR-R | RT-PCR | 5’-ACGACAACACGCAGTAACAAC-3’ |
